# Supplementary material for: Theta rhythmicity governs human behavior and hippocampal signals during memory-dependent tasks
Source: Nat Commun. 2021 Dec 2;12:7048. doi: 10.1038/s41467-021-27323-3 (PMC8639755; doi:10.1038/s41467-021-27323-3)
Supplement: Supplementary file 1 — Supplementary Information [file 41467_2021_27323_MOESM1_ESM.pdf]

Supplementary Information to:

Theta rhythmicity governs human behavior and hippocampal signals during memory-dependent tasks

Marije ter Wal[1,*], Juan Linde Domingo[1,2], Julia Lifanov[1], Frederic Roux[1], Luca D. Kolibius[1,3], Stephanie Gollwitzer[4], Johannes Lang[4], Hajo Hamer[4], David Rollings[5], Vijay Sawlani[5], Ramesh Chelvarajah[5], Bernhard Staresina[1,6], Simon Hanslmayr[1,3], Maria Wimber[1,3,*]

[1] School of Psychology & Centre for Human Brain Health, University of Birmingham, Edgbaston, B15 2TT, Birmingham, UK
[2] Max Planck Institute for Human Development, 14195, Berlin, Germany
[3] Centre for Cognitive Neuroimaging, School of Psychology and Neuroscience, University of Glasgow, G12 8QB, Glasgow, UK
[4] Universitätsklinikum Erlangen, 91054, Erlangen, Germany
[5] Complex Epilepsy and Surgery Service, Queen Elizabeth Hospital Birmingham, Edgbaston, B15 2GW, Birmingham, UK
[6] Department of Experimental Psychology, University of Oxford, OX2 6GG, Oxford, UK

*Correspondence: m.j.terwal@bham.ac.uk; maria.wimber@glasgow.ac.uk

Content

1. Supplementary Tables 1 – 8 pg ii - vi
2. Supplementary Figures 1 – 12 pg vii – xviii
3. Supplementary Note 1: Pairwise Phase Consistency for Macro electrodes pg xix
4. Supplementary Note 2: Simulated data for validation of O-score method pg xxi

1. Supplementary Tables 1 – 8

Supplementary Table 1. Demographic information for the 13 experiments included in this study. From left to right, the columns provide the following information: category = the general group the experiment fell under (refer to the main Methods section for more details); task version = number of the experiment used throughout the manuscript and stimulus set; # participant (excl.) = the number of participants that completed the experiment, with the number in brackets indicating the number of participants that were excluded due to poor performance for the catch questions; Avg. age ± SD = the mean age and standard deviation of all participants in years; sex = # number of males (m) and females (f) taking part in the study.

<table>
<tr><th>Category</th><th>Task version
& Stimulus set</th><th># part. (excl.)</th><th>Avg. age ± SD</th><th>Sex</th></tr>
<tr><td rowspan="4">Visual RT experiments (group 3)</td><td>1. Standard</td><td>23 (0 excl)</td><td>19.35 ± 1.11 yrs</td><td>19 f - 4 m</td></tr>
<tr><td>2. Standard - with background</td><td>24 (0 excl)</td><td>19.00 ± 0.88 yrs</td><td>20 f - 4 m</td></tr>
<tr><td>3. Shape</td><td>24 (0 excl)</td><td>18.71 ± 0.62 yrs</td><td>23 f - 1 m</td></tr>
<tr><td>4. Size</td><td>24 (0 excl)</td><td>19.04 ± 0.91 yrs</td><td>21 f - 3 m</td></tr>
<tr><td rowspan="5">Memory RT experiments (group 2)</td><td>5. Standard</td><td>26 (1 excl)</td><td>19.00 ± 0.80 yrs</td><td>23 f - 3 m</td></tr>
<tr><td>6. Standard with background</td><td>24 (4 excl)</td><td>19.50 ± 0.93 yrs</td><td>22 f - 2 m</td></tr>
<tr><td>7. Shape</td><td>25 (0 excl)</td><td>20.64 ± 2.36 yrs</td><td>17 f - 8 m</td></tr>
<tr><td>8. Size</td><td>23 (1 excl)</td><td>19.13 ± 0.90 yrs</td><td>23 f - 1 m</td></tr>
<tr><td>9. Standard - multiple retrievals</td><td>57 (5 excl)</td><td>19.95 ± 0.79 yrs</td><td>45 f - 12 m</td></tr>
<tr><td rowspan="4">Memory electrophys. experiments (group 1)</td><td>10. Standard - EEG</td><td>24 (0 excl)</td><td>21.91 ± 4.68 yrs</td><td>20 f - 4 m</td></tr>
<tr><td>11. Standard - EEG/fMRI</td><td>37 (1 excl)</td><td>23.31 ± 3.95 yrs</td><td>26 f - 11 m</td></tr>
<tr><td>12. Standard - iEEG image cue</td><td>3 (0 excl)</td><td rowspan="2">34.4 ± 9.11 yrs</td><td rowspan="2">5 f - 5 m</td></tr>
<tr><td>13. Standard - iEEG verb cue</td><td>7 (0 excl)</td></tr>
</table>

Supplementary Table 2. Task details for the different experiments. From left to right, the columns provide the following information: task # = number of the experiment used throughout the manuscript; type = experiment type (beh. = behavioral only); task phases = the task phases this experiment contributed to; Stimulus set = the stimulus set that was used; # trials = the number of unique objects (for visual tasks) or cue-object pairs (for memory tasks) the participants were presented with. For some of the visual tasks, the objects were shown twice, indicated by a 'x 2'; # retrieval repetitions (for memory tasks only; NA means not applicable) = the number of times each learned object had to be reinstated during the retrieval phases of the experiment; Catch questions: catch questions used for the experiment, with perc. = perceptual, sem. = semantic and cont. = contextual; # catch Q per trial = the number of catch questions that were asked after reinstatement.

Task #	Type	Task phases	Stimulus set	# trials	# ret. reps.	Catch questions:	# catch Q per trial
1	Beh.	Visual	Standard	128 x 2	NA	*Perc.:* drawing/photo<br>*Sem.:* animate/inanimate	1
2	Beh.	Visual	Standard background	128	NA	*Perc.:* drawing/photo<br>*Sem.:* animate/inanimate<br>*Cont.:* indoor/outdoor	1
3	Beh.	Visual	Shape	128 x 2	NA	*Perc.:* round/elongated<br>*Sem.:* natural/manmade	1
4	Beh.	Visual	Size	128 x 2	NA	*Perc.:* big/small<br>*Sem.:* natural/manmade	1
5	Beh.	Encoding<br>Catch-with-ret.	Standard	128	2	*Perc.:* drawing/photo<br>*Sem.:* animate/inanimate	1
6	Beh.	Encoding<br>Catch-with-ret.	Standard background	128	1	*Perc.:* drawing/photo<br>*Sem.:* animate/inanimate<br>*Cont.:* indoor/outdoor	1
7	Beh.	Encoding<br>Catch-with-ret.	Shape	128	2	*Perc.:* round/elongated<br>*Sem.:* natural/manmade	1
8	Beh.	Encoding<br>Catch-with-ret.	Size	128	2	*Perc.:* big/small<br>*Sem.:* natural/manmade	1
9	Beh.	Encoding<br>Catch-with-ret.	Standard	128	6<br>(7th & 8th excluded)	*Perc.:* drawing/photo<br>*Sem.:* animate/inanimate	1
10	EEG	Encoding<br>Retrieval<br>Catch-after-ret.	Standard	128	1	*Perc.:* drawing/photo<br>*Sem.:* animate/inanimate	2<br>(1 for 3 part.)
11	EEG<br>fMRI	Encoding<br>Retrieval<br>Catch-after-ret.	Standard	128	2	*Perc.:* drawing/photo<br>*Sem.:* animate/inanimate	1
12	iEEG	Encoding<br>Retrieval<br>Catch-after-ret.	Standard - image cue	64	1	*Perc.:* drawing/photo<br>*Sem.:* animate/inanimate	1
13	iEEG	Encoding<br>Retrieval<br>Catch-after-ret	Standard - verb cue	128	1	*Perc.:* drawing/photo<br>*Sem.:* animate/inanimate	2

Supplementary Table 3. Reaction time descriptors for correct trials per task phase, across all included participants. Source data are provided as a Source Data file.

Measure	Task phase				
	**Encoding**	**Retrieval**	**Catch-with-retrieval**	**Catch-after-retrieval**	**Visual**
Mean RT (s)	3.99	2.20	2.24	1.42	0.89
SD of RT (s)	2.57	2.22	1.26	1.25	0.45
5% bound (s)	1.29	0.68	0.97	0.53	0.44
95% bound (s)	6.81	5.15	4.79	3.22	1.67
Mean # responses per participant	66.27	150.68	217.66	158.17	215.20
SD # responses per participant	33.93	54.28	99.2	57.88	54.1

Supplementary Table 4. Post-hoc tests for Z-scored O-scores from the Oscillation score procedure. Abbreviations: Bonf.-corr. = Bonferroni-correction; t-stat. = t-statistic; Df = degrees of freedom.

**Group(s)**	**Comparison**	**Test (two-tailed)**	**Bonf.-corr.**	**t-stat.**	**Df.**	**p-value**
Group 1	Encoding vs Retrieval	Paired t-test	3	-0.76	63	1.00
	Encoding vs Catch-after-retrieval	Paired t-test	3	1.98	64	0.155
	Retrieval vs Catch-after-retrieval	Paired t-test	3	2.46	68	0.0495
Group 2	Encoding vs Catch-with-retrieval	Paired t-test	NA	-0.94	116	1.00
Group 1 & Group 2 vs Group 3	Encoding vs Visual	Two-sample t-test	4	7.23	274	< 0.001
	Retrieval vs Visual	Two-sample t-test	4	5.83	161	< 0.001
	Catch-with-retrieval vs Visual	Two-sample t-test	4	6.48	236	< 0.001
	Catch-after-retrieval vs Visual	Two-sample t-test	4	4.04	162	0.0034

Supplementary Table 5. Effect sizes of the O-scores per task phase (i.e., against the permuted baseline), and between task phases (i.e., comparing against each other).

	**d'**		**Cohen's d**				
			**Encoding**	**Retrieval**	**Catch-with-ret.**	**Catch-after-ret.**	**Visual**
**Encoding**	0.46			-0.21	-0.12	0.23	0.92
**Retrieval**	0.56				0.064	0.39	0.92
**Catch-with-retrieval**	0.43					0.28	0.86
**Catch-after-retrieval**	0.20						0.64
**Visual**	-0.42						

Supplementary Table 6. Post-hoc tests for peak frequency from the Oscillation score procedure. Z-stat. = Z-statistic.

Group(s)	Comparison	Test (all two-tailed)	Bonferroni-correction	Z-Stat.	p-value
Group 1	Encoding vs Retrieval	Wilcoxon signed rank	3	1.081	0.839
	Encoding vs Catch-after-retrieval	Wilcoxon signed rank	3	-1.197	0.694
	Retrieval vs Catch-after-retrieval	Wilcoxon signed rank	3	-1.776	0.227
Group 2	Encoding vs Catch-with-retrieval	Wilcoxon signed rank	NA	-3.201	0.0014
Group 1 & Group 2 vs Group 3	Encoding vs Visual	Wilcoxon rank-sum	4	-4.529	<0.001
	Retrieval vs Visual	Wilcoxon rank-sum	4	-3.664	<0.001
	Catch-with-retrieval vs Visual	Wilcoxon rank-sum	4	-2.593	0.038
	Catch-after-retrieval vs Visual	Wilcoxon rank-sum	4	-1.340	0.721

Supplementary Table 7. Implantation information per patient: the number of Behnke-Fried microwire bundles that were implanted in hippocampus (in brackets the number of bundles that were located outside of hippocampus) and the number of functional hippocampal wires we recorded from. In the right column, the referencing and re-referencing scheme is indicated.

**Patient ID**	**# microwire bundles**	**# functional hippocampal wires**	**References**
01	4 (+ 2 removed due to artifacts	29	Low impedance wires
02	6	48	High impedance wires
03	6	42	Ground, re-referenced to bundle mean
04	4	32	Low impedance wires
05	5 (1)	40	Low impedance wires
06	6 (2)	48	Low impedance wires
07	5	39	Low impedance wires
08	2	16	Low impedance wires
09	2	16	Low impedance wires
10	2	16	Low impedance wires
**Total:**	**42 (3)**	**326**	

Supplementary Table 8. Task and recording information per patient, as well as the number of trials that were included in the analyses.

**Patient ID**	**Task version**	**# sessions**	**# correct encoding tr**	**# incorrect encoding tr**	**# correct retrieval tr**	**# incorrect retrieval tr**
01	12. Image cue	1	48	14	50	14
02	12. Image cue	1	55	9	55	9
03	12. Image cue	1	61	3	61	2
04	13. Verb cue	2	99	27	101	20
05	13. Verb cue	2	33	9	66	14
06	13. Verb cue	3	90	31	92	31
07	13. Verb cue	3	107	18	108	15
08	13. Verb cue	2	116	12	116	12
09	13. Verb cue	1	42	21	43	20
10	13. Verb cue	2	103	25	103	25

2. Supplementary Figures 1 – 10

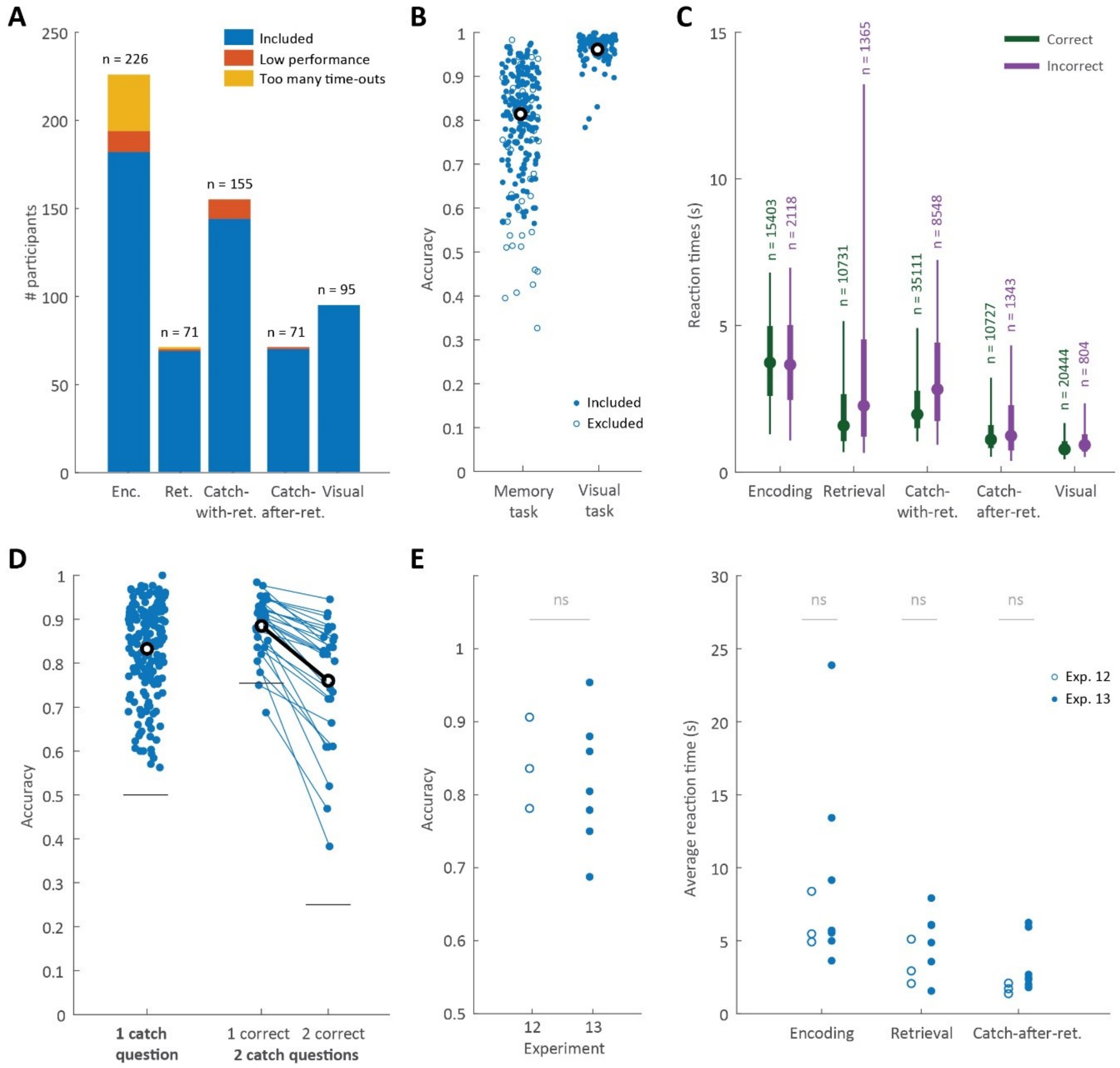

Supplementary Figure 1. Descriptors of behavioral data. **A**: Number of included participants (blue) and the number of participants excluded for bad performance (orange) or insufficient number of data points due to trial time-outs (yellow) per task phase. Numbers above each bar indicate the total number of participants that were recorded. Enc. is Encoding; Ret. is Retrieval; Catch-with. is Catch-with-retrieval; **B**: Accuracy per participant on the memory and visual tasks. Included participants are indicated by filled circles, excluded participants by open circles; **C**: Box plots showing the reaction time distributions, across all button presses from all participants, for correct (green) and incorrect trials (purple), per task phase. Box plots indicate the 5, 25, 50 (circles), 75 and 95% boundaries; **D**: Fraction of correct trials for memory tasks with 1 catch question asked after retrieval (left), or two questions asked (middle and right). For participants answering two questions (connected dots), we show the fraction of trials with at least 1 question answered correctly (middle) and the fraction with two questions correct (right). Horizontal lines represent guessing level; **E**: Task performance (left) and reaction times (right) of the iEEG patients who participated in experiments 12 (open circles) and 13 (filled circles). Statistical comparison using a two-tailed t-test (α=0.05); ns is not significant. Source data are provided as a Source Data file.

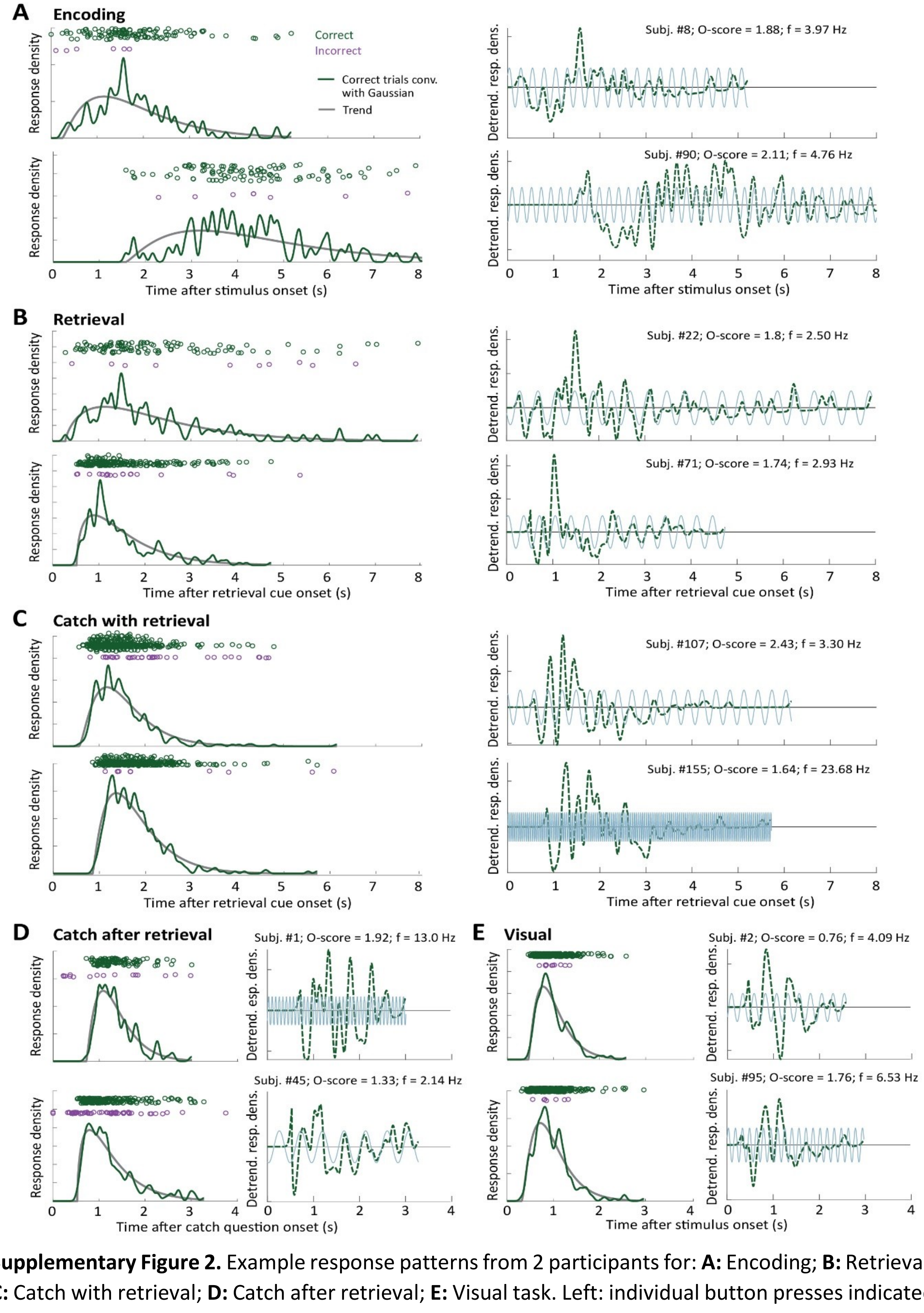

Supplementary Figure 2. Example response patterns from 2 participants for: **A:** Encoding; **B:** Retrieval; **C:** Catch with retrieval; **D:** Catch after retrieval; **E:** Visual task. Left: individual button presses indicated by dots (green for correctly remembered and purple for (later) forgotten trials). The green line shows the correct responses smoothed with a Gaussian kernel and the gray line the fitted trend curve. Right: The difference between these, i.e. the 'detrended response density' (dashed green line), fitted with a sine wave (light blue) at the dominant frequency identified by the O-score (given above the plot). Source data are provided as a Source Data file.

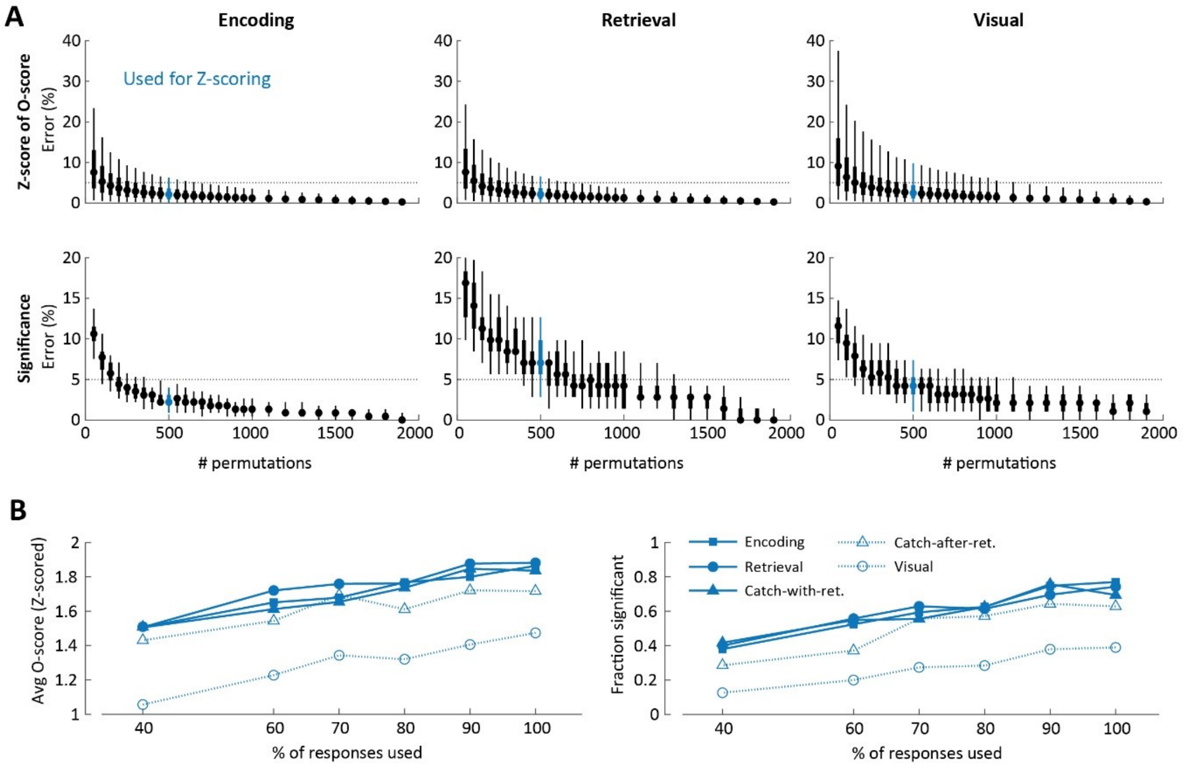

Supplementary Figure 3. Validation of parameters of the O-score analyses. **A**: To test the Z-scoring method for the O-score, we computed reference distributions with 2000 permutations for each participant for Encoding (left), Retrieval (middle) and Visual (right) task phases, and down-sampled the reference distribution to between 50 and 1900 permutations (x-axes), each repeated 50 times. We report the deviation (%) relative to the Z-scores obtained for 2000 permutations (top row) and the percentage difference in the number of participants marked as significant (bottom row). The number of permutations used throughout the manuscript (500 permutations), is indicated in blue. Horizontal dashed lines indicate a 5% error. Box plots indicate the 5, 25, 50 (circles), 75 and 95% boundaries of the distribution across the 50 down-sampling repetitions; **B**: Average Z-scored O-score (left) and fraction of participants with a significant O-score (right) as a function of the percentage of responses used to analyze the O-score. Reducing the percentage of responses included in the O-score analysis reduced the O-score and the fraction of significant participants, but this reduction affected all 5 task phases in a similar way. Task phases are indicated by line/symbol combinations: solid line with filled squares: Encoding; solid line with filled circles: Retrieval; solid line with filled triangle: Catch-with-retrieval; dashed line with open triangle: Catch-after-retrieval and dashed line with open circle: Visual. Source data are provided as a Source Data file.

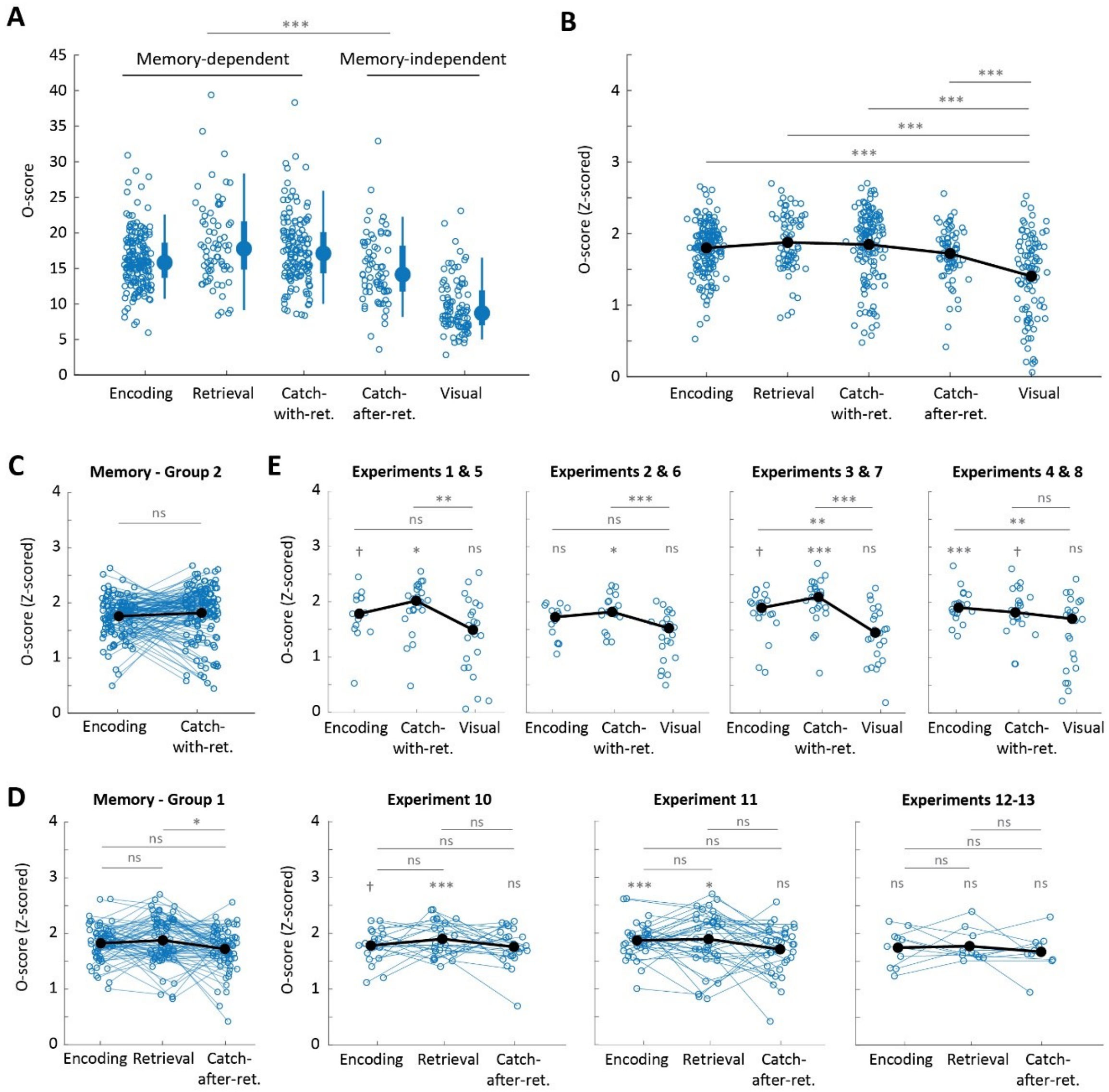

Supplementary Figure 4. Additional O-score analyses. **A**: Raw O-scores per task phase. Each dot is one participant. Box plots (right) summarize the data across participants and show the 5, 25, 50 (circles), 75 and 95% boundaries; **B**: t-tests between Visual task phase and all other task phases (two-tailed, α=0.05, Bonferroni corrected for 4 comparisons); **C**: Paired t-test between task phases for memory task group 1 (two-tailed, α = 0.05). Connected dots are from one participant; **D**: Paired t-test between task phases for memory task group 2 (two-tailed, α=0.05, Bonferroni corrected for 3 comparisons); **E**: Data split into stimulus set and experiments (see Supplementary Tables 1 and 2), with the data from groups 2 and 3 in the top row and the data from group 1 in the bottom row. Symbols directly above the data indicate the group-level statistics, analogous to Figure 3A. For the top row, the data were further compared between the memory task phases (group 2) and the visual task (group 3) using t-tests (two-tailed, α=0.05, Bonferroni corrected for 2 comparisons); For the bottom row, the data were compared using paired-tests (two-tailed, α=0.05, Bonferroni corrected for 2 comparisons). Connected dots are from one participant and black lines and dots shows the median O-scores; ns: not significant; †: p≤0.05 before correction; after multiple comparisons correction: *: 0.05≥p>0.01; **: 0.01≥p>0.001; ***: p≤0.001. Source data are provided as a Source Data file.

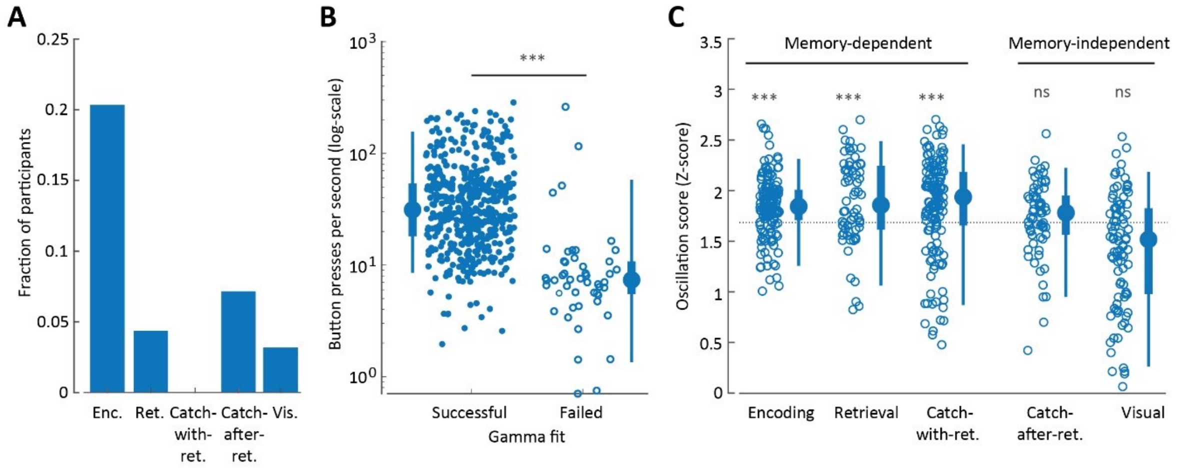

Supplementary Figure 5. Characterization of the gamma trend-fit used to generate reference distributions for Z-scoring of the O-scores. **A:** Fraction of participants included in the O-scores procedures for which the gamma fit failed. This fraction was high for the Encoding button presses, likely caused by the low number of responses and long response times (see Supplementary Table 3 and B), but low for other task phases. For the participants where the fit failed, a reference distribution was instead generated by shuffling the response times randomly, i.e., without maintaining the overall trend in the responses (giving rise to the data shown in Figure 3 A and B). **B**: Participants and task phases for which the gamma fit failed (open circles) had a substantially lower response density (i.e., number of button presses per second, please note the log scale), than datasets with successful (filled circles) gamma fits (two-tailed two-sample t-test; $t(558) = 4.48$; $p<0.001$). Each circle represents the response density from one participant and task phase, and box plots represent the 5, 25, 50, 75 and 95% bounds of the distribution across participants and task phases. **C:** Scatter plot of O-scores (Z-scored) per task phase only using participants for which the gamma trend curve was successful. Each circle is one participant, and box plots represent the 5, 25, 50, 75 and 95% bounds of the O-score distribution across participants. The dashed line gives the significance threshold for single participants ($\alpha=0.05$, one-tailed, Z-distribution). Qualitatively similar results were obtained when excluding participants for whom a gamma fit failed, compared to the results reported in the main text (compare with the data shown in Figure 3A). Statistical comparisons only minimally differed when using this smaller sample, with second level statistics as follows: Encoding: $t(144)=7.89$; $p<0.001$; Retrieval: $t(65)=4.63$; $p<0.001$; Catch-with-retrieval: $t(143)=5.09$; $p<0.001$; Catch-after-retrieval: $t(64)=1.62$; $p=0.277$; Visual: $t(91)=-3.93$; $p=1.00$; all Bonferroni-corrected for 5 comparisons. These statistics are also given above each task phase: ns: not significant; *: $0.05\geq p>0.01$; **: $0.01\geq p>0.001$; ***: $p\leq 0.001$, Bonferroni-corrected for 5 comparisons. Source data are provided as a Source Data file.

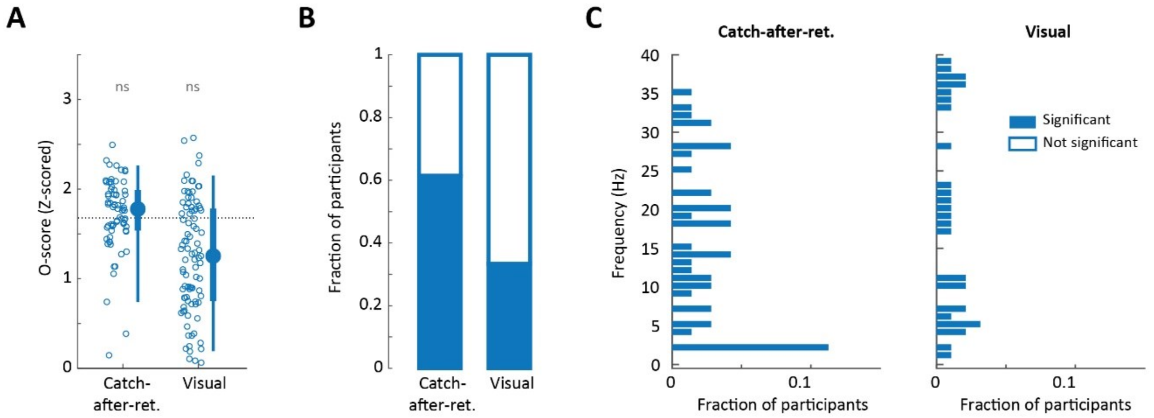

Supplementary Figure 6. Control analysis for lower frequency boundary of O-score procedure for the Catch-after-retrieval and Visual task phases. The lower frequency boundary was reduced from a maximum period of 1/3 of the time series to 2 times the time series' length (i.e., 6 times lower). Z-scored O-scores (**A**), fraction of participants with significant O-scores (**B**) and frequency distributions for Catch-after-retrieval and Visual task phases (**C**) are shown using this lenient lower frequency boundary. Statistical comparison of the O-score as in the main text. ns: not significant. Source data are provided as a Source Data file.

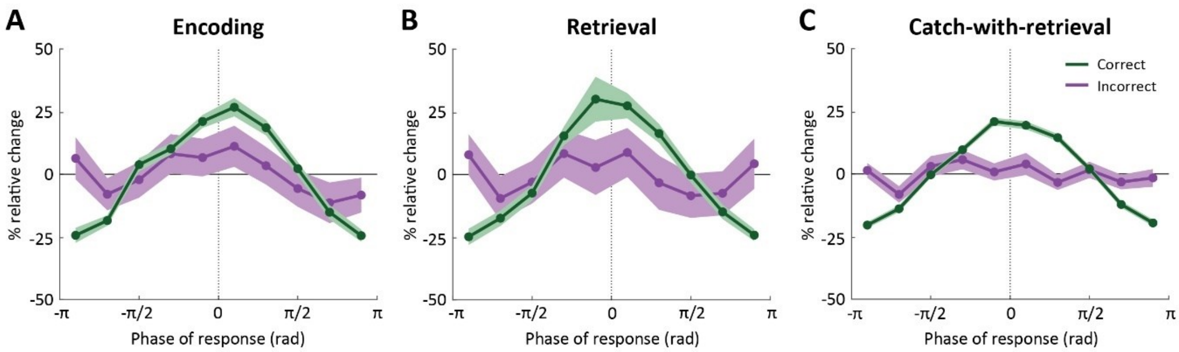

Supplementary Figure 7. Phase distribution of down-sampled set of correct (green) and all incorrect (purple) trials relative to the phase trace determined based on all remaining correct trials of the same participant for Encoding (**A**), Retrieval (**B**) and Catch-with-retrieval button presses (**C**). Only participants with a sufficient number of incorrect trials (at least 10) were included. The number of correct trials was down-sampled to the number of incorrect trials. All remaining correct trials were used to determine the phase trace, which in turn was used to determine the phase-of-response of the down-sampled correct and incorrect trials. Subsampling was repeated 100 times per participant and the phase distribution was determined across these repetitions. Lines with dots indicate the mean across participants and the shaded areas in the figure show the standard error of the mean. These data correspond to the data shown on the right in Figure 4 A-C; for statistics see Figure 4 and main text. Source data are provided as a Source Data file.

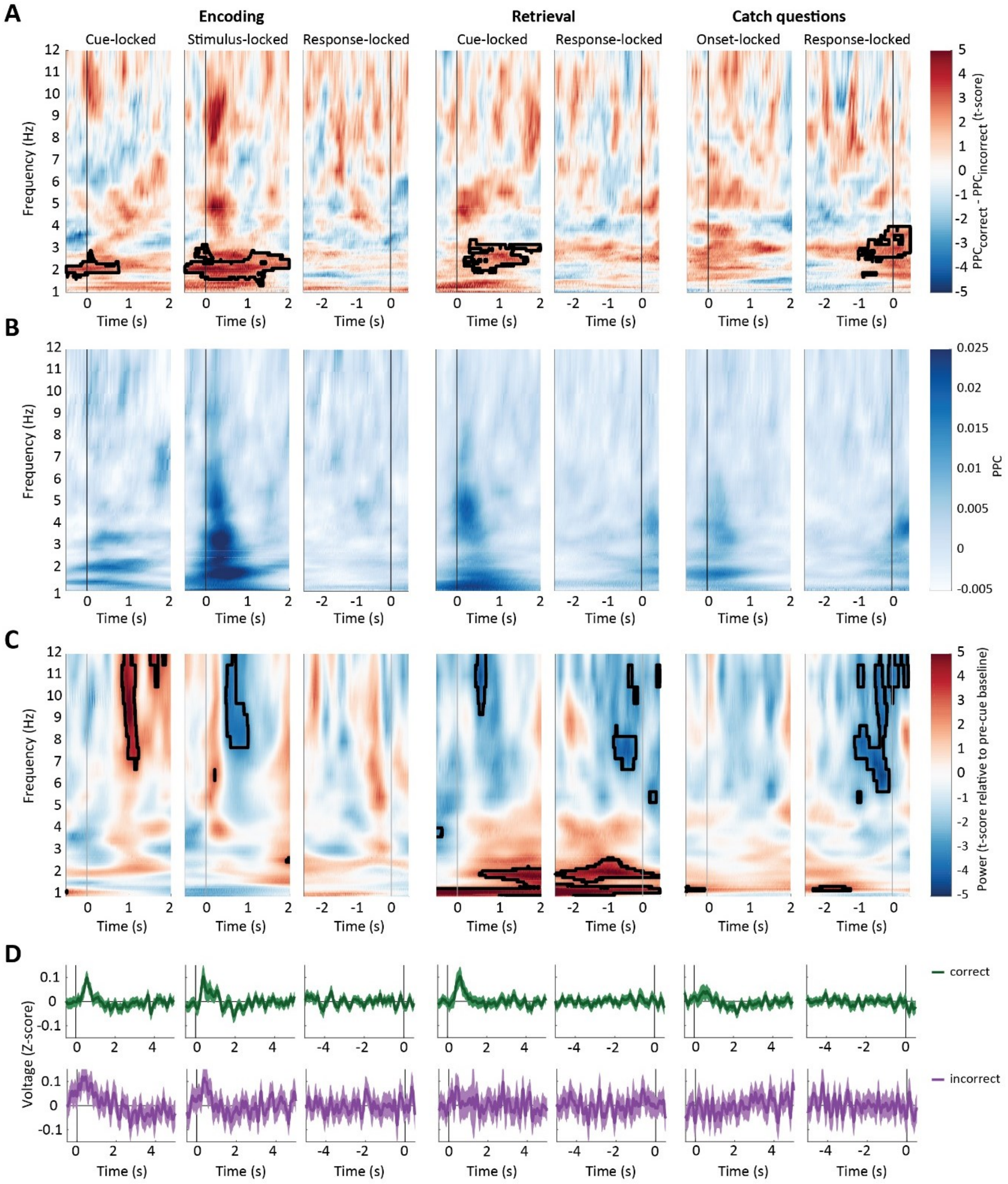

Supplementary Figure 8. Supplementary data for Pairwise Phase Consistency (PPC) analyses on microwire data from Figure 5. **A**: PPC for correct versus incorrect trials (t-scored, color-coded). Black outlines indicate significance (α=0.05) compared to a reference distribution with permuted trial-labels; **B**: Averaged raw PPC values for correct trials across all subjects. Significant PPC increases (see Figure 5B) are accompanied by increases in raw PPC; **C**: Average power changes relative to baseline for correct trials across all subjects; **D**: Event-related potentials (ERPs) for correct (green, top row) and incorrect trials (purple, bottom row). Voltage traces were Z-scored per trial relative to pre-cue baseline and averaged across trials, channels and subjects. Solid lines show the smoothed (50 ms kernel) mean voltage and shaded areas indicate the standard error of the mean. All panels show data from encoding trials (left, cue-, stimulus- and response-locked), retrieval trials (middle, cue- and response-locked) and catch questions (onset- and response-locked), with vertical black lines indicating t = 0 s.

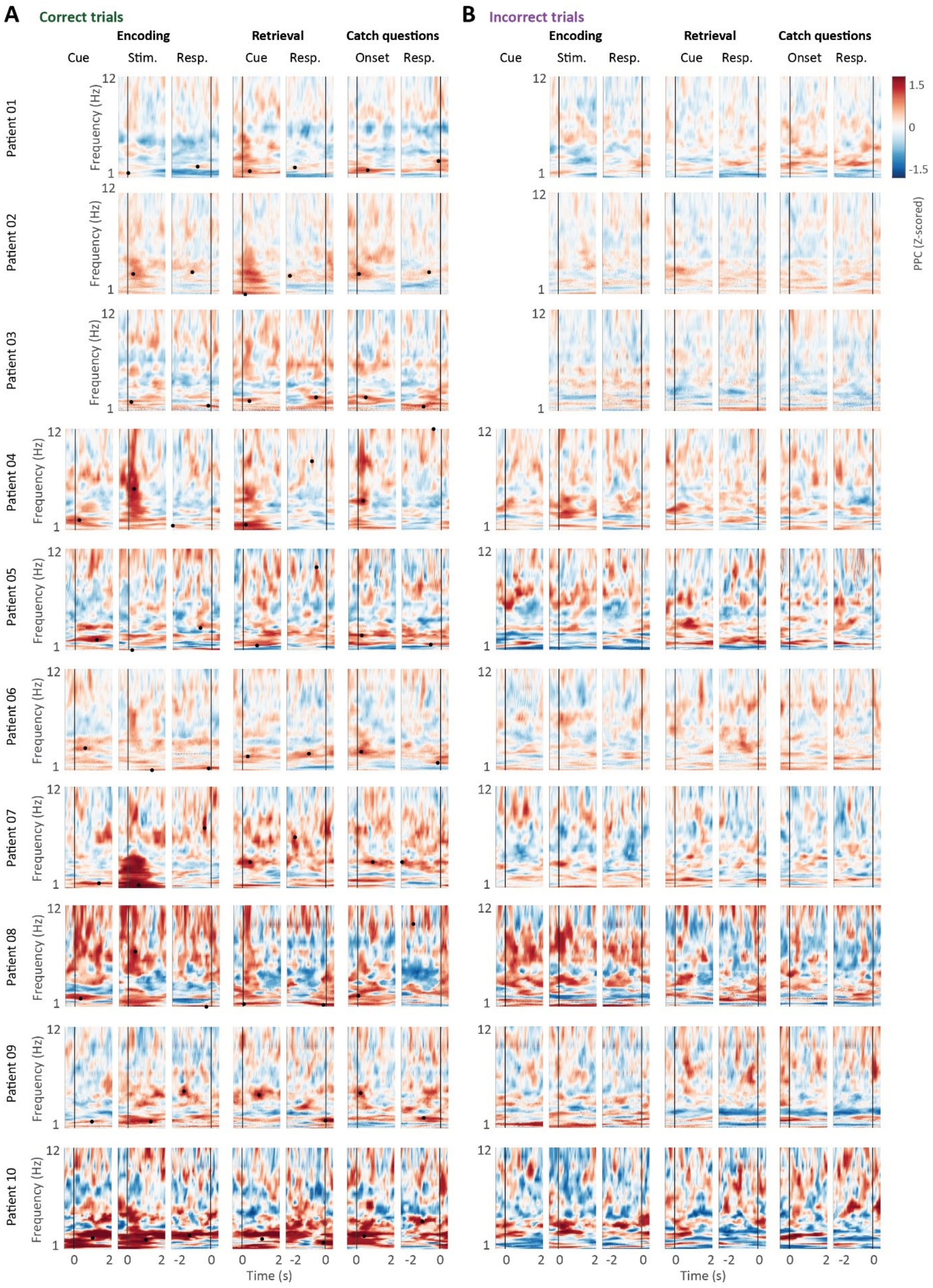

Supplementary Figure 9. Average Pairwise Phase Consistency (PPC), Z-scored relative to pre-cue baseline (color-coded) for the microwire data from each of the 10 intracranial EEG patients for correct (**A**) and incorrect trials (**B**), and locked to cue onset, stimulus onset and response for encoding trials, cue onset and response for retrieval trials, as well as for catch question onset and response.

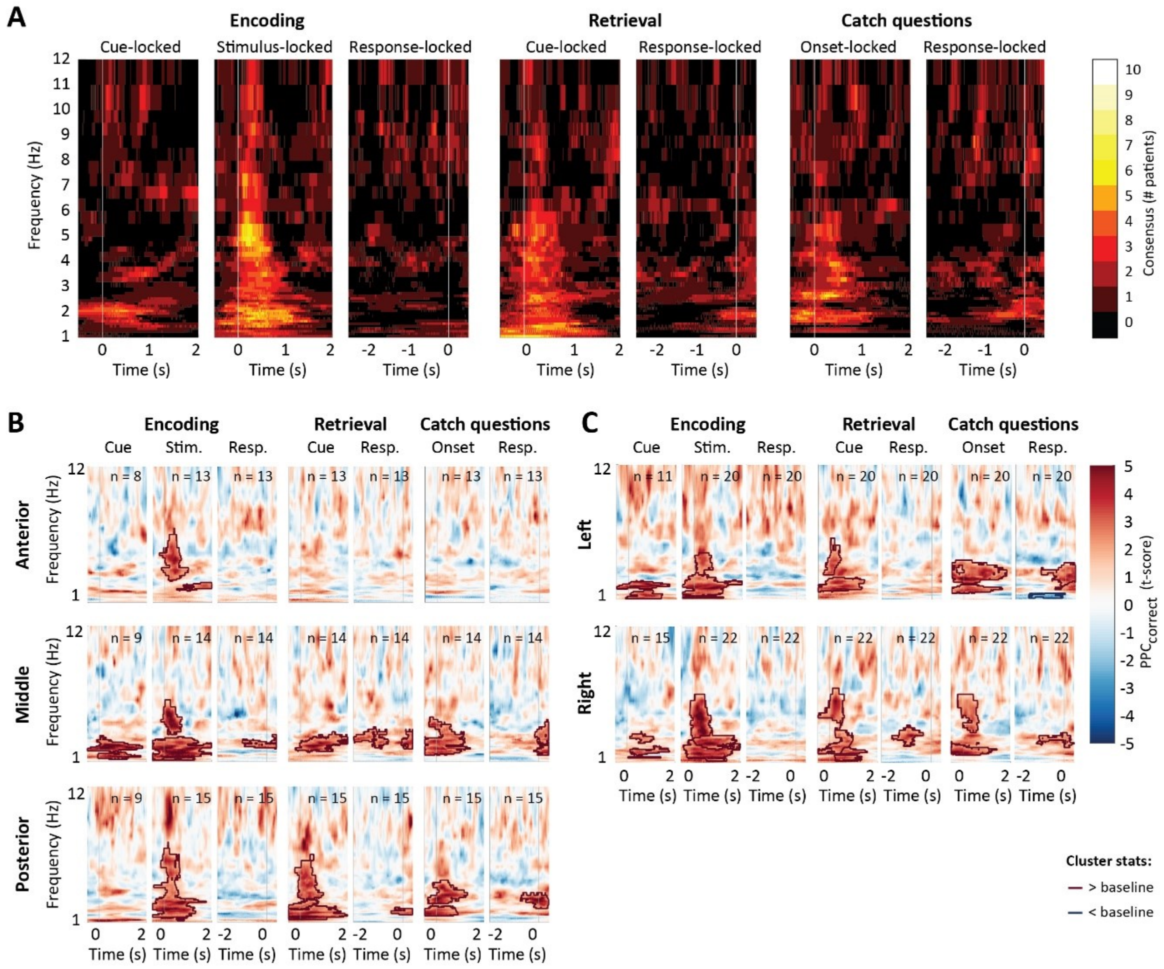

Supplementary Figure 10. A: Consensus analysis for microwire PPC of correct trials. Histogram of the number of patients that reached significant PPC (α=0.05, uncorrected) against the patient-specific reference PPC distribution obtained from time-shuffled trials; **B**: Showing the same data as in Figure 5B of the main text, microwire PPC of correct trials are shown for anterior, middle and posterior hippocampus, color code shows the second level t-score. Outlines indicate significant changes (red = increases; blue = decreases) relative to baseline, as compared to a time-shuffled dataset. Number of electrode bundles included is shown in every panel; **C**: as H, showing the same data split into left and right hemisphere.

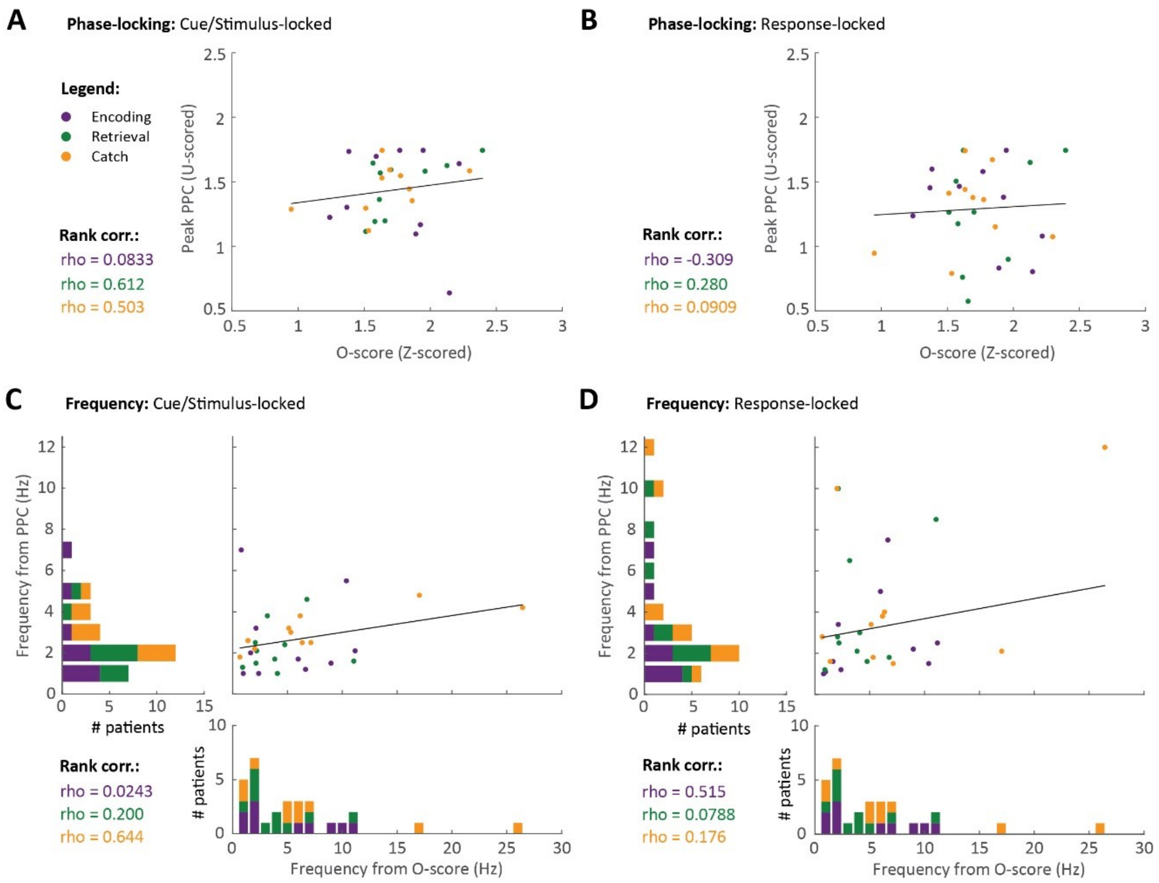

Supplementary Figure 11. Comparison of O-score analyses with peak PPC values and frequencies. **A&B**: Scatter plots showing O-scores (horizontal axes) versus peak PPC values for the 10 intracranial EEG patients obtained for encoding (purple), retrieval (green) and catch questions (yellow). Data are shown for both cue/stimulus onset-locked analyses (**A**) and response-locked analyses (**B**). The Spearman rank correlation is given for each task phase separately in the same color code. Note that caution is required when interpreting these cross-participant correlations, due to the relatively low number of data points. **C&D**: As A&B, but showing the peak frequencies identified by the O-score (horizontal axes) and the peak PPC (vertical axes). To aid comparison of the frequencies, histograms are shown for O-score peak frequencies (vertical bars) and peak PPC frequencies (horizontal bars). Source data are provided as a Source Data file.

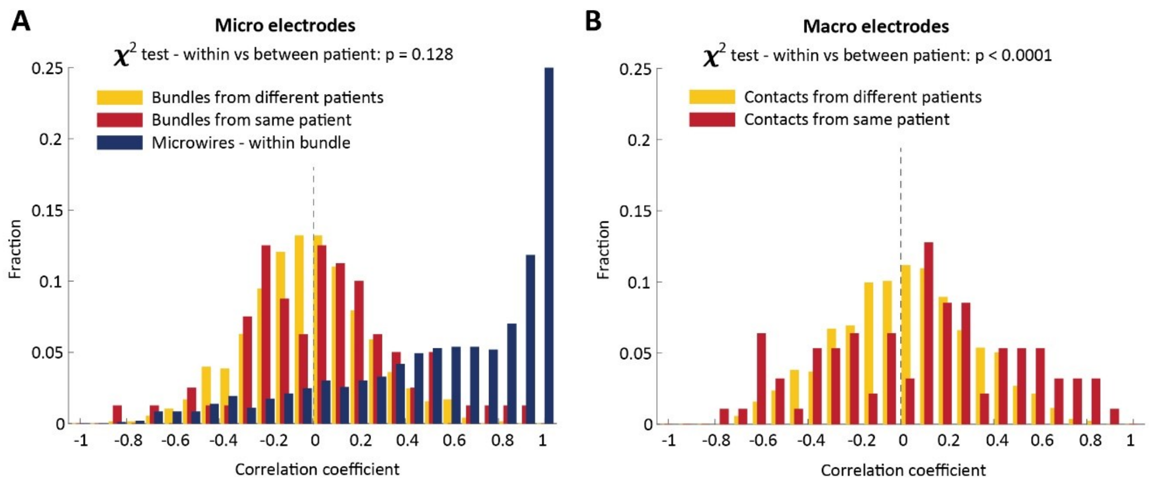

Supplementary Figure 12. Dependencies of ERPs in the 2 seconds after stimulus onset during encoding, for correct trials, from different electrodes within and between subjects. **A:** ERPs of different microwires and averages within microwire bundles were correlated with each other, to identify similarities between wires from the same bundle (blue), bundles from the same subject (red) and bundles from two different subjects. Here the correlation coefficient distributions are shown as histograms: The x-axis shows the correlation coefficient in bins, the y-axis the fraction of the total number of pairs of microwires/bundles. The dashed gray line indicates a correlation coefficient of 0. Different wires within a bundle showed strong correlations. Averages of wires within bundles were not strongly correlated (i.e., centered around 0), and this was the case for when the bundles were from different patients or from the same patient. Correlation coefficient distributions of bundles from the same and different patients did not significantly differ from each other (χ^2-test; $\chi^2(25) = 33.14$; $p = 0.128$). Data from the Behnke-Fried microwire electrodes thus indicate that bundle is the main source of variance; **B:** As A, but for macro contacts. Correlation coefficients for macro contacts from two different patients (yellow) centered around 0, while correlations between contacts from the same patient (red) were slightly higher, resulting in a significant difference between the two distributions (χ^2-test; $\chi^2(25) = 103.21$; $p<0.001$), thus suggesting patient as the main source of variance.

3. Supplementary Note 1: Pairwise Phase Consistency for Macro electrodes

For comparison with other studies, we repeated the Pairwise Phase Consistency (PPC) analysis presented in Figure 5, for the macro electrode contacts in hippocampus for the same patients. Analyses were performed in a way that was identical to that described for the micro electrodes in the Methods section of the main text, with one important difference: the second level analysis was performed at the subject level for the signals from the macro contacts, while this was done at the bundle level for the signals from the microwires. This choice was made as it was established that the ERPs from micro electrode bundles were as independent from each other within subjects as between subjects, but this could not be established for the macro contacts (see Supplementary Figure 12). The PPC results from the macro contacts were therefore first averaged within subjects, before performing the second level statistics. The results are shown in Supplementary Figure 13.

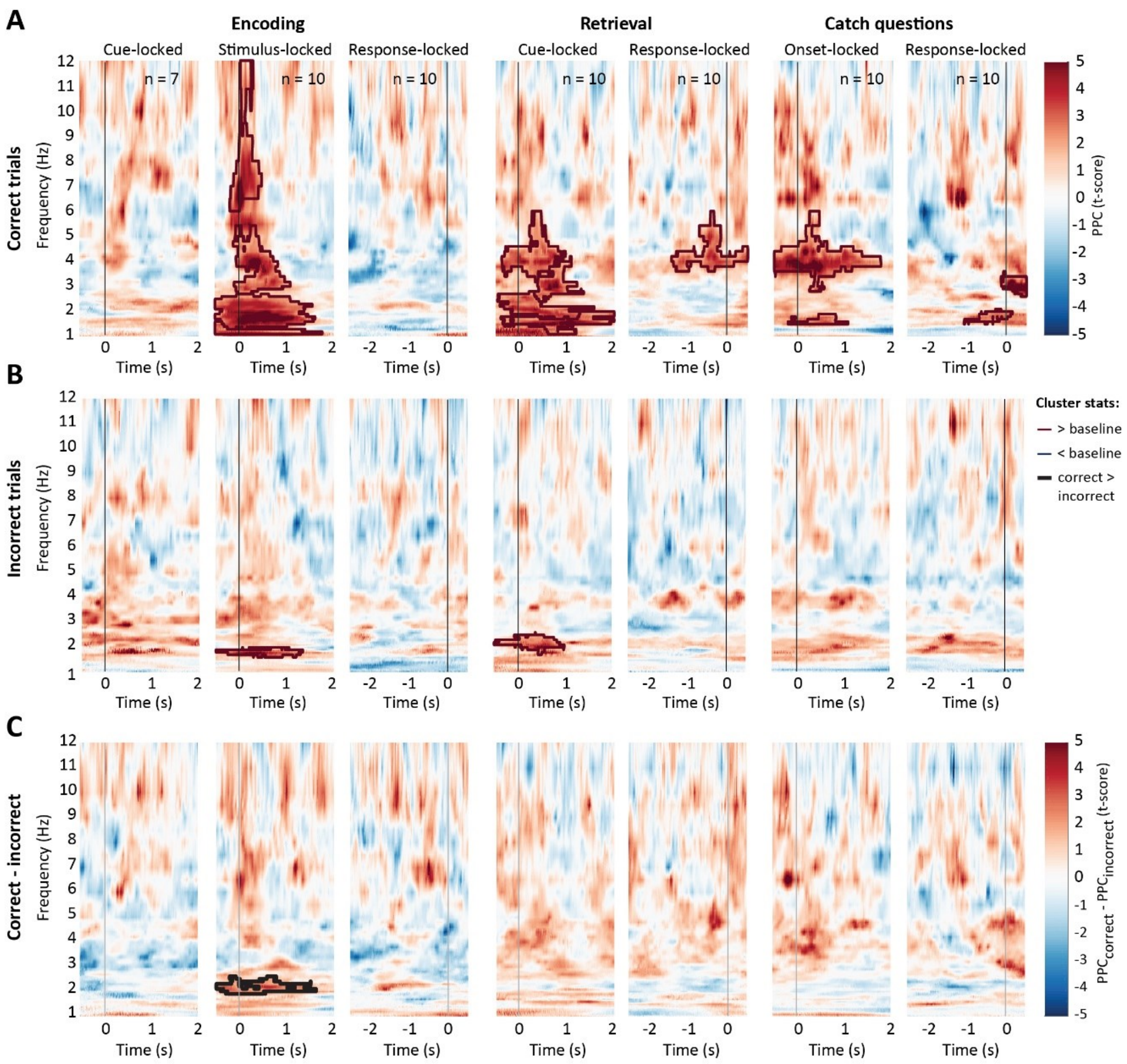

Supplementary Figure 13. Pairwise phase consistency across trials (PPC, color-coded, second level t-score) for macro electrodes, analogous to the analysis of micro electrodes shown in Figure 5. Analysis was performed separately for correct trials (A) and incorrect trials (B), and the contrast between correct and incorrect trials (C), locked to cue/stimulus onset or response of encoding (left column), retrieval (middle) and catch trials (right). Significant changes from baseline (α=0.05, permutation test against time-shuffled trials) are indicated separately for increases (red) and decreases (blue). Black outlines indicate significant differences between correct and incorrect trials (α=0.05, permutation test against shuffled trial labels). Second level analyses were performed on the patient-level; the number of datapoints are given in A, for each task phase, and also apply to B and C.

4. Supplementary Note 2: Simulated data for validation of O-score method

To test the applicability and performance of the O-score method for our data, we ran a series of simulations. In these simulations, we produced model data with and without oscillations, i.e., with a known ground truth, and computed O-scores in the same way as reported for the behavioral data. This allowed us to test whether the O-score method is able to detect oscillations of different strengths in a noisy dataset, as well as reject data with no oscillation included. In addition, it allowed us to compare the O-score's ability to detect oscillations for datasets with different characteristics, such as average response times, with each other. To this end, we modelled data that matched the overall response distributions, the number of responses per participant and task phase, and the number of included participants from the encoding and visual task phases.

Response data were simulated as a Poisson process, i.e.:

$$P_{\text{resp}}(t \to t + \Delta t) = r(t)\,\Delta t \tag{1}$$

with P_{resp} the chance of a response occurring at the time interval t to $t + \Delta t$, $r(t)$ the 'firing rate' at time t and Δt the time step, set to Δt = 0.0005 s. The firing rate function $r(t)$ was modelled as:

$$r(t) = N^{s}_{\text{spikes}}\, r^{s}_{\text{trend}}(t)\, r_{\text{osc}}(t) \tag{2}$$

i.e., as the product of the total number of spikes N_{spikes}, an overall trend function $r_{\text{trend}}(t)$ and an oscillatory function $r_{\text{osc}}(t)$, of which the former two factors were varied between model participants s, introducing noise in the data set. The total number of spikes was drawn from a normal distribution, the mean and SD of which were matched by the behavioral data (Supplementary Table 3) and with a minimum of 10 spikes.

For the visual task phase, the firing rate function $r_{\text{trend}}(t)$ was modelled using a gamma probability density function, while the trend for the encoding task phase was modelled using a normal probability density function and the retrieval task phase with a lognormal distribution. The length of the time series was varied between model participants and was drawn from a uniform distribution. The parameters for the probability density functions are given in Supplementary Table 9.

To introduce the oscillation, we used:

$$r_{\text{osc}}(t) = A \sin\,(2\pi\, f_{\text{osc}}\, t) \tag{3}$$

with f_{osc} the frequency of the oscillation, which was set to 2.5, 5, 7.5, 10 and 15 Hz, and A the amplitude, varying between 0 (i.e., no oscillation) and 1 (i.e. 100% modulation of response rate within each period), in steps of 0.1. The results for a range of f_{osc} and A is given for both model task phases in Supplementary Figure 14.

Supplementary Table 9. Parameters of the model data. See Supplementary Methods for details.

**Task phase:**	**# model participants**	**# spikes Mean ± SD:**	**$r_{\text{trend}}(t)$:**					
			**Pdf function**	**Parameter 1**		**Parameter 2**		**Total time (s)**
Encoding	190	66 ± 34	Normal	Mean:	1.5-2.5	St dev:	2.5-3.5	4-12 s
Retrieval	70	151 ± 54	Lognormal	Mean:	0-1	St dev:	1-1.5	4-12 s
Visual	95	215 ± 54	Gamma	Shape:	1-2	Scale:	0.25-0.5	1.5-4.5 s

In summary, we simulated data resembling the encoding, retrieval and visual task phases in terms of the number of participants, reaction time distributions and number of responses per participant, with various levels of oscillatory modulation. Some example simulations are given in Supplementary Figure 14A. Identical to the analyses on the observed data, we computed the O-score for each simulated participant. To aid comparison with Figure 3 of the main text, we report the distributions of O-scores across participants (mean and standard deviation given in the top rows of Supplementary Figure 14B-D), and the number of participants reaching the significance threshold (bottom rows of Supplementary Figure 14B-D). We also computed second level statistics and report whether the results are significant at $\alpha = 0.01$ (corrected). Finally, we computed frequency histograms across significant O-scores (middle rows of Supplementary Figure 14B-D).

The results in Supplementary Figure 14 provide us with two important validations of the O-score method. Firstly, we observe that O-scores were low and did not reach significance when no or weak oscillatory modulations were applied, and this was the case for each of the 3 task phases. This suggests that the O-score analysis is not likely to provide spurious results when an oscillation is not present or is too weak to be detected.

Conversely, for stronger oscillations, O-scores became significant for each of the three task phases, and the fraction of participants with significant O-scores went up markedly. Importantly, when the O-score analysis indicated significance at the population level, then the peak frequencies were overwhelmingly detected at the correct frequency (red dotted lines in Supplementary Figure 14 indicate a 2 Hz frequency band around the ground truth frequency). The O-score method therefore is very specific, both in terms of detecting an oscillation only when one is present in the data, and in detecting the correct frequency at which this oscillation occurred.

The oscillation amplitude at which the significance threshold was reached differed per task phase, with a low threshold of 20-30% modulation sufficient for Retrieval and a high 50-60% required for Encoding. This differentiation is expected given the large differences in response density between the task phases, with extremely low response densities for Encoding (i.e., few responses over a long timeframe). We note that O-scores remained non-significant and the fraction of significant participants was low when the oscillation frequency was expected to be undetectable given the characteristics of the data set, again suggesting spurious results are unlikely. This occurred for the 15 Hz oscillation for Encoding, due to low response density and corresponding reduction in the upper frequency bound. O-scores also dropped for low oscillation frequencies for the Visual task, due to the short response timeframe and corresponding increase in lower frequency bound. The latter drop in O-score could be 'rescued' by increasing the maximum detectible oscillation period (i.e., minimum frequency) for the standard 1/3rd of the timeframe to 2 times the response timeframe (yellow Supplementary Figure 14D), confirming the validity of the analyses shown in Supplementary Figure 6.

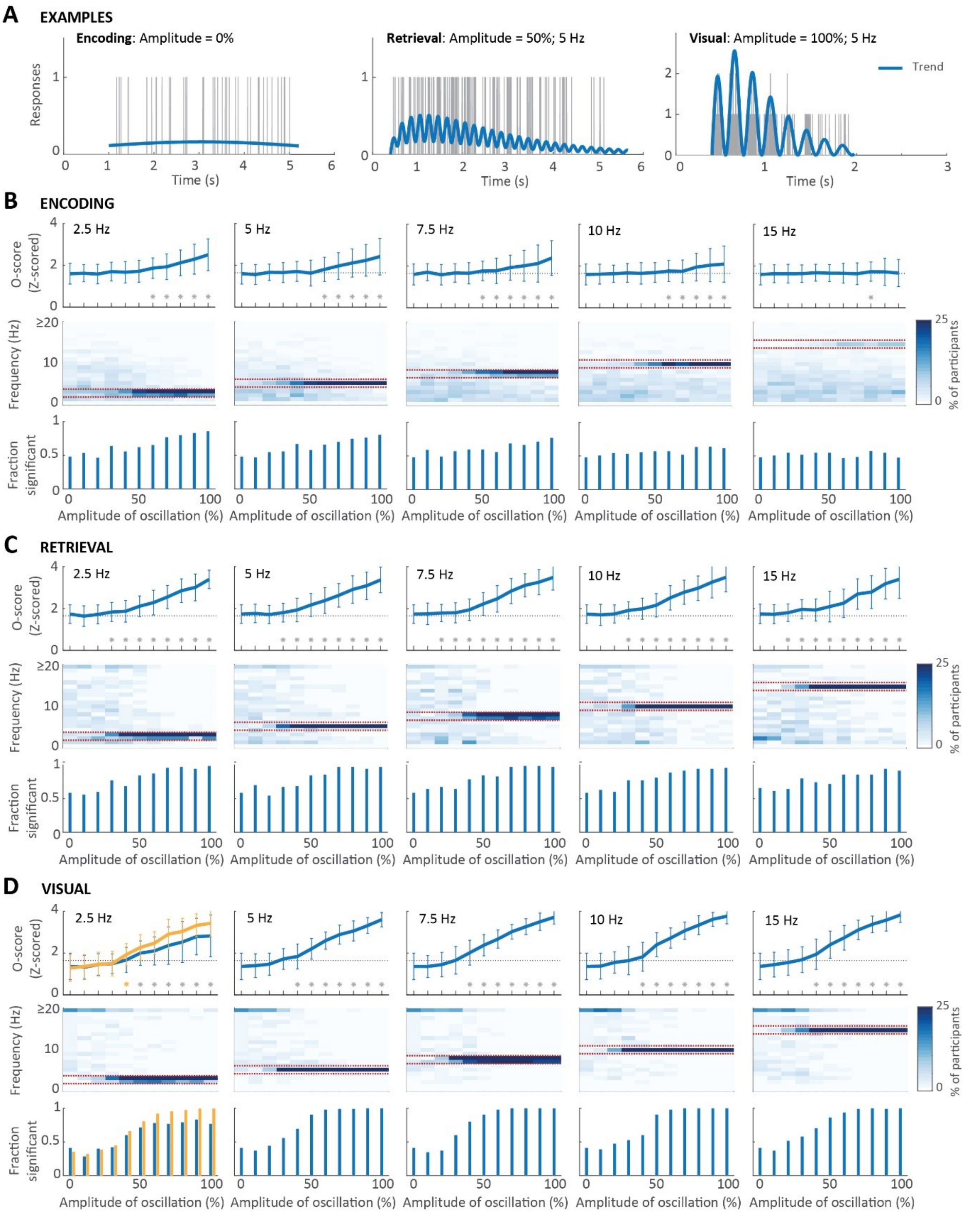

Supplementary Figure 14. O-scores for simulated data for Encoding (B), Retrieval (C) and Visual (D), for a range of oscillation amplitudes (x-axes; 0% = no oscillation; 100% = complete modulation), and 5 frequencies (left to right): 2.5, 5, 7.5, 10 and 15 Hz. **A**: Example traces: trend curves in blue and response traces in grey. **B-D**: Per task phase, we show the Z-scored O-score (top row, mean ± standard deviation, *: significance for population at α = 0.01), frequency histogram per amplitude (middle row, color-coded, y-axis truncated at 20 Hz) and fraction of participants with significant O-scores (bottom row). In D, an additional simulation is shown for 2.5 Hz, were the lower frequency bound for O-score computation was reduced by a factor 6 (yellow). For all task phases, O-scores were only significant when oscillations were present (top rows) and then correctly identified their frequency (middle rows). Source data are provided as a Source Data file.
