# Supplementary material for: Theta rhythmicity governs human behavior and hippocampal signals during memory-dependent tasks
Source: Nat Commun. 2021 Dec 2;12:7048. doi: 10.1038/s41467-021-27323-3 (PMC8639755; doi:10.1038/s41467-021-27323-3)
Supplement: Supplementary file 3 — Reporting summary [file 41467_2021_27323_MOESM3_ESM.pdf]

nature research

Corresponding author(s): Marije ter Wal

Last updated by author(s): Sep 30, 2021

Reporting Summary

Nature Research wishes to improve the reproducibility of the work that we publish. This form provides structure for consistency and transparency in reporting. For further information on Nature Research policies, see our Editorial Policies and the Editorial Policy Checklist.

Statistics

For all statistical analyses, confirm that the following items are present in the figure legend, table legend, main text, or Methods section.

n/a	Confirmed	
☐	☒	The exact sample size (n) for each experimental group/condition, given as a discrete number and unit of measurement
☐	☒	A statement on whether measurements were taken from distinct samples or whether the same sample was measured repeatedly
☐	☒	The statistical test(s) used AND whether they are one- or two-sided<br>*Only common tests should be described solely by name; describe more complex techniques in the Methods section.*
☐	☒	A description of all covariates tested
☐	☒	A description of any assumptions or corrections, such as tests of normality and adjustment for multiple comparisons
☐	☒	A full description of the statistical parameters including central tendency (e.g. means) or other basic estimates (e.g. regression coefficient) AND variation (e.g. standard deviation) or associated estimates of uncertainty (e.g. confidence intervals)
☐	☒	For null hypothesis testing, the test statistic (e.g. F, t, r) with confidence intervals, effect sizes, degrees of freedom and P value noted<br>*Give P values as exact values whenever suitable.*
☒	☐	For Bayesian analysis, information on the choice of priors and Markov chain Monte Carlo settings
☐	☒	For hierarchical and complex designs, identification of the appropriate level for tests and full reporting of outcomes
☐	☒	Estimates of effect sizes (e.g. Cohen's d, Pearson's r), indicating how they were calculated

Our web collection on statistics for biologists contains articles on many of the points above.

Software and code

Policy information about availability of computer code

Data collection	All tasks were presented and logged using MATLAB 2015a-2018a (The Mathworks), with Psychophysics Toolbox Version 3 (https://github.com/Psychtoolbox-3/Psychtoolbox-3). Intracranial EEG data were recorded using an ATLAS recording setup running Cheetah software v1.1.0 (Neuralynx Inc.).
Data analysis	Data analysis was performed using MATLAB version 2018a (The Mathworks), using the Wavelet toolbox (v5.0) and Statistics and Machine Learning Toolbox (v11.3). The following external toolboxes were used: CircStats toolbox 2012a (https://github.com/circstat/circstat-matlab); FieldTrip v20190615: https://github.com/fieldtrip/fieldtrip; NeuralynxImportExport v6.0.0: https://neuralynx.com/software/category/matlab-netcom-utilities; SPM12: https://www.fil.ion.ucl.ac.uk/spm/. Electrode localization was performed using MRIcron v1.0.20190902 (https://people.cas.sc.edu/rorden/mricron/index.html) and visualization was done in ModelGUI release 1.0.30: http://www.modelgui.org. The MATLAB code used for data analysis is available on: https://github.com/marijeterwal/behavioral-oscillations (https://doi.org/10.6084/m9.figshare.13213769).

For manuscripts utilizing custom algorithms or software that are central to the research but not yet described in published literature, software must be made available to editors and reviewers. We strongly encourage code deposition in a community repository (e.g. GitHub). See the Nature Research guidelines for submitting code & software for further information.

Data

Policy information about availability of data

All manuscripts must include a data availability statement. This statement should provide the following information, where applicable:

- Accession codes, unique identifiers, or web links for publicly available datasets
- A list of figures that have associated raw data
- A description of any restrictions on data availability

All behavioral data and the PPC data presented in the manuscript are available via the following figShare repository: https://doi.org/10.6084/m9.figshare.c.5192567. Requests for other data, such as intermediate processing steps or derived iEEG data that are not presented in the paper can be addressed to the corresponding author (m.j.terwal@bham.ac.uk), but raw iEEG data and information that would allow for identification of individual participants (such as patient specific electrode locations), cannot be provided due to consent and privacy restrictions.

Field-specific reporting

Please select the one below that is the best fit for your research. If you are not sure, read the appropriate sections before making your selection.

☒ Life sciences ☐ Behavioural & social sciences ☐ Ecological, evolutionary & environmental sciences

For a reference copy of the document with all sections, see nature.com/documents/nr-reporting-summary-flat.pdf

Life sciences study design

All studies must disclose on these points even when the disclosure is negative.

Sample size	Sample sizes for the behavioral studies were predetermined based on the original research question they were designed to address; the relevant considerations are described in Linde-Domingo et al., 2019, referenced in the Methods section. The sample size of the iEEG dataset was deemed sufficient prior to the start of data analysis based on the existing literature and a good coverage of bilateral hippocampus (> 40 electrodes).
Data exclusions	Participant's behavioral data were excluded when their memory performance was indistinguishable from chance level and/or if the number of data points was low (<10). These exclusion criteria were set a priori and are described in the Results section and the Methods section of the paper. For the iEEG data, recording channels and/or time points containing epileptic activity, movement artifact or electrical artifact were excluded, in line with common practice. These procedures were designed before data analysis started and are described in the Methods section.
Replication	We included two different versions of the memory task in the paper (group 1 and group 2), which differed in the design of the retrieval phase, as they originally aimed to accommodate electrophysiological recordings and direct comparison with the visual task, respectively. Each group contained several different experiments, with slightly different experimental designs and/or stimulus sets: the memory task was repeated in a total of 9 experiments and the visual task in a total of 4 experiments. These groups and experiments function as internal replications for the main behavioral effects reported, producing qualitatively and quantitatively similar results, as shown in Figures 3 and 4 and Supplementary Figure S4. For the PPC analyses, data from individual patients, as well as a consensus analysis, and data split into different hemispheres and subregions of the hippocampus are included in the Supplementary Information to demonstrate consistency across patients/regions. The results were also replicated in the same patients using recordings from the macro contacts.
Randomization	Healthy participants were recruited for either the visual task or the memory task to comply with consent and ethics regulations. To avoid differences between groups, the same inclusion criteria were used for both tasks (normal or corrected vision; no history of neurological or psychiatric disorders). Furthermore, the same communication channels were used to recruit participants, and data were recorded in the same facilities (with the exception of the participants in the MRI experiment). Demographics of the groups are reported below and in the Supplementary Information. All patients performed the same task, so randomization was not applicable here.
Blinding	All participants and experimenters had a basic understanding of the aim of the study, but all were unaware of the research question and data analysis approach presented in this manuscript during data collection. MtW and MW were aware of the hypothesis whilst analyzing the data. Participants and data collectors were not blinded to the group (memory or visual) they were assigned to, as this knowledge was essential for instructing (for data collectors) and for consenting to and performing (for participants) the task (the groups performed different tasks, see Figure 1).

Reporting for specific materials, systems and methods

We require information from authors about some types of materials, experimental systems and methods used in many studies. Here, indicate whether each material, system or method listed is relevant to your study. If you are not sure if a list item applies to your research, read the appropriate section before selecting a response.

Materials & experimental systems

n/a	Involved in the study
x	Antibodies
x	Eukaryotic cell lines
x	Palaeontology and archaeology
x	Animals and other organisms
	x Human research participants
x	Clinical data
x	Dual use research of concern

Methods

n/a	Involved in the study
x	ChIP-seq
x	Flow cytometry
x	MRI-based neuroimaging

Human research participants

Policy information about studies involving human research participants

Population characteristics	Demographics were as follows:<br>exp = experiment; f = female, m = male; yrs = years<br><br>Visual tasks:<br>- exp 1: 19 f - 4 m; 19.35 +- 1.11 yrs<br>- exp 2: 20 f - 4 m; 19.00 +- 0.88 yrs<br>- exp 3: 23 f - 1m; 18.71 +- 0.62 yrs<br>- exp 4: 21 f - 3 m; 19.04 +- 0.91 yrs<br><br>Memory group 2:<br>- exp 5: 23 f- 3 m; 19.00 +- 0.80 yrs<br>- exp 6: 22 f - 2 m; 19.50 +- 0.93 yrs<br>- exp 7: 17 f - 8 m; 20.64 +- 2.36 yrs<br>- exp 8: 23 f - 1 m; 19.13 +- 0.90 yrs<br>- exp 9: 45 f - 12 m; 19.95 +- 0.79 yrs<br><br>Memory group 1:<br>- exp 10: 20 f - 4 m; 21.91 +- 4.68 yrs<br>- exp 11: 26 f - 11 m; 23.32 +- 3.95 yrs<br><br>Patients:<br>- exp 12 & 13: 5 f - 5 m; 34.4 +- 9.11 yrs<br><br>Healthy participants reported normal or corrected to normal vision and no history of neurological of psychiatric disorders.
Recruitment	Healthy participants were recruited using the research participation scheme of the University of Birmingham (SONA Systems) and given a choice between university course credits or a cash payment (£6-8 per hour) as compensation for their time. Compensation was independent of task performance. Epilepsy patients were recruited by the clinical teams; patients received no compensation and were informed that participation would not benefit their clinical treatment. No details about the specific research questions addressed here, or the data analysis plan, were provided during recruitment to healthy participants and patients, limiting the potential effect of self-selection biases. Furthermore, several of the presented analyses compared data within participants, such as retrieval versus catch question button presses, or correct versus incorrect responses, further reducing the impact of any biases.
Ethics oversight	All studies involving healthy participants were approved by the Science, Technology, Engineering and Mathematics Ethical Review Committee of the University of Birmingham. The work with epilepsy patients was approved by the National Health Service Health Research Authority (15/WM/2019), the Research Governance & Ethics Committee from the University of Birmingham, and the Ethik-Kommission der Friedrich-Alexander Universität Erlangen-Nürnberg (142_12 B).

Note that full information on the approval of the study protocol must also be provided in the manuscript.
